# Effects of curcumin on the bioavailability of dioxin-like pollutants in rats

**DOI:** 10.1038/s41598-021-92085-3

**Published:** 2021-06-14

**Authors:** Delei Cai, Qing Chen, Jianlong Han, Yanhua Song, Zhen Meng, Yibin Zheng, Haitao Shen

**Affiliations:** Zhejiang Provincial Center for Disease Control and Prevention, Binsheng Road 3399, Hangzhou, 310051 China

**Keywords:** Environmental sciences, Natural hazards

## Abstract

The effects of curcumin on the bioavailability of polychlorinated dibenzo-*p*-dioxins/furans (PCDD/Fs) and dioxin-like polychlorinated biphenyls (DL-PCBs) were investigated in Sprague–Dawley rats. Tetra- and penta-chlorinated PCDFs had the lowest bioavailability and hexa-chlorinated PCDD/Fs had the highest, while there was no obvious change in that of DL-PCBs. Curcumin markedly reduced the toxic equivalent (TEQ) of PCDD/Fs in rats, illustrating the potential to competitively inhibit absorption of PCDD/Fs by the epithelial cells of the small intestine due to the similar chemical structure (diphenyl) between curcumin and PCDD/Fs. Moreover, curcumin lowered the TEQ of DL-PCBs in the liver of male rats, but not female rats. The significant decrease in the bioavailability of PCDD/Fs and DL-PCBs demonstrates the potential detoxification mechanisms of curcumin.

## Introduction

Polychlorinated dibenzo-*p*-dioxins/furans (PCDD/Fs), also known as dioxins, and dioxin-like polychlorinated biphenyls (DL-PCBs) are typical persistent organic pollutants (POPs). The main health hazards of DL-POPs to humans include neurotoxicity, reproductive toxicity, hepatotoxicity, endocrine disruption, thyroid disruption, and carcinogenicity^1^. Much attention has been paid to the burdens and toxicological effects of DL-POPs to animals, the environment, and food crops^2^.

Studies have reported that dietary intake accounts for 90% of POP exposure to humans^3^. Considering the great harm of POPs to human health, it is important to prevent the intake and accumulation, and promote the excretion of POPs to reduce toxicological effects. The efficacies and mechanisms of bioactive extracts as potential antidotes to PCDD/Fs have been widely researched. For example, substances with antioxidant activity, such as docosahexaenoic acid, have protective effects against 2,3,7,8-tetrachlorodibenzo-*p*-dioxin (TeCDD)-induced hepatotoxicity^4^. *Chlorella pyrenoidosa* can facilitate the excretion of 1,2,3,4,7,8-hexachlorodibenzo-*p*-dioxin (HpCDD) in feces and reduce its accumulation in the mouse liver^5^. Administration of mineral oil, activated carbon, and rice bran can alleviate the toxic effects of dioxins^6^.

**Figure 1:**
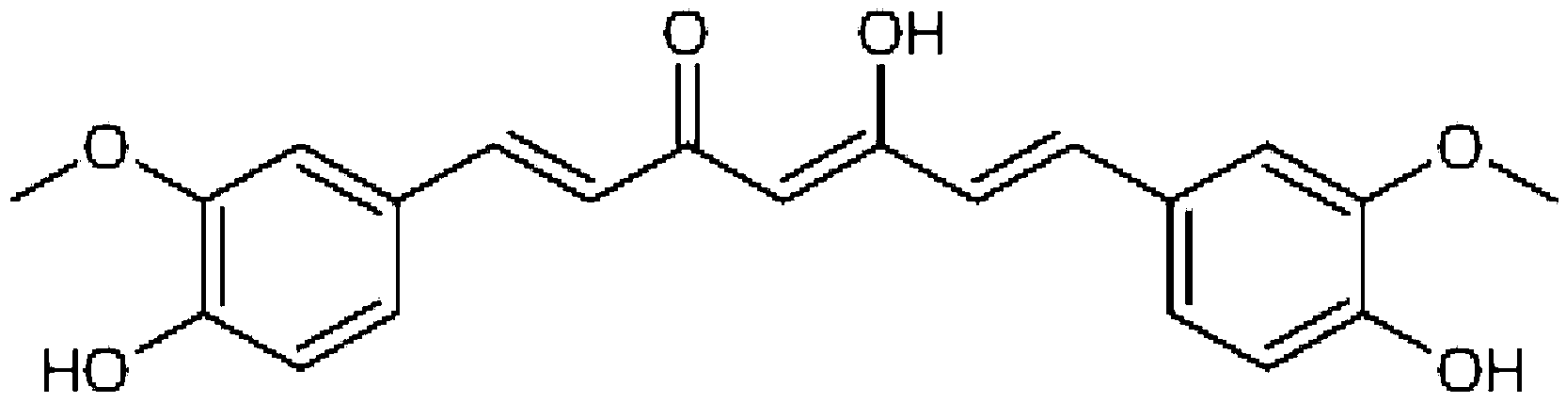
Chemical structure of curcumin.

Curcumin, the active ingredient of the traditional herbal remedy and spice turmeric (*Curcuma longa*), contains phenolic and quinone groups that exert protective effects against oxidative stress, humoral immunosuppression, and gastric mucosal injury in rats induced by 2,3,7,8-TeCDD^7^. It is currently believed that the mechanism of curcumin that promotes the detoxification of 2,3,7,8-TeCDD is mainly realized through aryl hydrocarbon receptor (AhR)-mediated pathways^8^. Analyses of curcumin homologues and quantitative structure–activity relationships have shown that keto-enol tautomerism of the methoxy group and β-diketone structure of curcumin is essential to inhibit AhR transformation (Fig. 1), while the addition of methyl and methoxy groups can enhance the inhibitory effects of curcumin^9^. As a possible alternative pathway underlying the detoxification of DL-POPs, curcumin is reported to decrease the absorption of DL-POPs in the epithelium of the small intestine, which leads to reduced bioavailability and decreased exposure to organisms, thus, exerting a detoxifying effect. To verify this hypothesis, the aim of the present study was to investigate the effects of curcumin on the bioavailability of PCDD/Fs and DL-PCBs in the rat liver and to identify possible mechanisms of curcumin underlying the detoxification of DL-POPs.

## Materials and methods

The study protocol was approved by the Animal Care Committee of Zhejiang Provincial Center for Disease Control and Prevention (ZJCDC), and conducted in accordance with the ZJCDC Animal Care and Animal Research: Reporting of In Vivo Experiments guidelines.

Twelve DL-PCBs and 10 PCDD/Fs (see Supplementary S-1) were purchased from AccuStandard, Inc. (New Haven, CT, USA). Curcumin standards were obtained from Zhejiang Medicine Co., Ltd. (Shaoxing, China). Weights were measured with an electronic balance (PL1001-L; Mettler Toledo, Columbus, OH, USA) and compounds were analyzed with a high-resolution gas chromatography system (6890N; Agilent Technologies, Palo Alto, CA, USA) coupled with a high-resolution mass spectrometer (HRMS; Autospec Ultima; Micromass UK Ltd., Manchester, UK).

In total, 32 Sprague–Dawley (SD) rats (16 males and 16 females) were obtained from Shanghai Laboratory Animal Co., Ltd. (Shanghai, China), housed at a constant temperature of 20–24 °C and relative humidity of 50–70%, and fed standard rat chow (Zhejiang Laboratory Animal Center, Hangzhou, China) for 4 days before the experiments. After the acclimation period, the SD rats were randomly assigned to one of three groups: a control group (A), PCDD/Fs + DL-PCBs exposure group (B), or curcumin intervention group (C). The rats in each group were housed in separate cages with ad libitum access to food and water. The rats in group B were administered 5 mL/kg body weight (BW) of PCDD/Fs and DL-PCBs (diluted with soybean oil to final concentrations of 1 and 10 ng/mL) by daily oral gavage, in addition to 10 mL/kg BW of distilled water for 28 consecutive days. The rats in group C were administered the same doses of PCDD/Fs and DL-PCBs by oral gavage, in addition to 10 mL/kg BW of an aqueous solution of curcumin (0.13 g/kg BW) orally for 28 consecutive days. The rats in group A were administered 5 mL/kg BW of soybean oil and 10 mL/kg BW of distilled water (S1–3). The BW and food intake were recorded once weekly. After the last administration, all animals were fasted overnight, anesthetized with isoflurane, and killed to obtain the liver, which was stored at − 40 °C for later use.

$$\mathrm{B}\mathrm{i}\mathrm{o}\mathrm{a}\mathrm{v}\mathrm{a}\mathrm{i}\mathrm{l}\mathrm{a}\mathrm{b}\mathrm{i}\mathrm{l}\mathrm{i}\mathrm{t}\mathrm{y}\,\,\mathrm{w}\mathrm{a}\mathrm{s}\,\,\mathrm{c}\mathrm{a}\mathrm{l}\mathrm{c}\mathrm{u}\mathrm{l}\mathrm{a}\mathrm{t}\mathrm{e}\mathrm{d}\,\,\mathrm{a}\mathrm{c}\mathrm{c}\mathrm{o}\mathrm{r}\mathrm{d}\mathrm{i}\mathrm{n}\mathrm{g}\,\,\mathrm{t}\mathrm{o}\,\,\mathrm{t}\mathrm{h}\mathrm{e}\,\,\mathrm{f}\mathrm{o}\mathrm{r}\mathrm{m}\mathrm{u}\mathrm{l}\mathrm{a}:\,\,\mathrm{B}A=\frac{C\times M}{10\times c\times V}$$

where BA is the bioavailability (%) of PCDD/Fs or DL-PCBs in the liver, C is the detected concentration (pg/g) of PCDD/Fs or DL-PCBs in the liver, M is the weight (g) of the whole liver, c is the intragastrical concentration (ng/mL) of PCDD/Fs or DL-PCBs, and V is the total intragastrical volume (mL) on day 28.

All data are presented as the mean ± standard deviations. Normality of variable distribution was verified using the Shapiro–Wilk and David–Hellwig tests. The independent samples Student’s *t*-test was used to compare values between two groups. A probability (*p*) value of ≤ 0.05 was considered statistically significant. The effect of a single factor on the measurement results was assessed using one-way analysis of variance (ANOVA). If there were no significant differences between the groups, no further tests were performed. If the null hypothesis was rejected by ANOVA (*p* < 0.05), the significance of differences between group means was determined using the Student–Newman–Keuls post hoc test for multiple comparisons. If the distribution of the studied parameter did not conform to the assumptions of ANOVA, the Kruskal–Wallis test was used as a nonparametric alternative. All analyses were performed using EXCEL (Microsoft Corporation, Redmond, WA, USA) and IBM SPSS Statistics for Windows, version 19.0. (IBM Corporation, Armonk, NY, USA).

## Results

**Figure 2:**
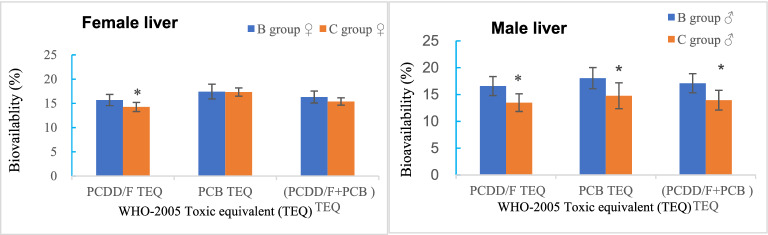
Effects of curcumin on bioavailability of WHO-TEQ 2005 (*p < 0.05) (drawn with Microsoft Excel).

There were no significant differences in BW on days 0, 14, and 28, empty stomach weight, and liver weight of female and male rats among groups A, B, and C (ANOVA, *p* > 0.05). However, there were significant differences in the hepatosomatic index (HIS; liver weight relative to BW) of male rats among the three groups (ANOVA, *p* < 0.05). Specifically, the HSI of male rats was markedly higher in group B than group A (Q test, *p* < 0.05) and notably lower in group C than group B (Q test, *p* < 0.05). No significant difference in the HSI of female rats was observed among the three groups (ANOVA, *p* > 0.05) (Table 1). The bioavailability of WHO-PCDD/F-TEQ, WHO-DL-PCB-TEQ, and total TEQ in the liver of male rats declined significantly in group C as compared to group B (*t*-test, *p* < 0.05). In the female rats in group C, the bioavailability of WHO-PCDD/F-TEQ in the liver was significantly lowered (*t*-test, *p* < 0.05), but there were no significant changes in the bioavailability of WHO-DL-PCB-TEQ or total TEQ (*t*-test, *p* > 0.05) (Fig. 2).

**Table 1:** Effects of PCDD/Fs, DL-PCBs, and curcumin on BW, liver weight, and HSI of rats ($\stackrel{-}{x}$± s, g).

Group	Sex	Initial BW	Mid-study BW	Final BW	Final empty stomach weight	Final liver weight	HSI (%)
A (n = 4)	Female	70.3 ± 2.6	158.0 ± 3.5	206.4 ± 3.4	183.6 ± 4.0	6.6 ± 0.4	3.6 ± 0.1
B (n = 4)		72.5 ± 3.2	158.2 ± 7.0	206.0 ± 13.1	183.1 ± 10.3	6.8 ± 0.3	3.7 ± 0.2
C (n = 8)		70.6 ± 1.7	154.5 ± 5.3	197.4 ± 7.7	181.3 ± 4.2	6.4 ± 0.6	3.6 ± 0.3
A (n = 4)	Male	72.4 ± 1.2	179.2 ± 13.6	288.0 ± 17.7	249.8 ± 17.7	8.8 ± 0.6	3.5 ± 0.1
B (n = 4)		72.8 ± 1.8	182.0 ± 4.0	288.2 ± 19.6	252.0 ± 15.8	9.8 ± 1.0	3.9 ± 0.2^**#**^
C (n = 8)		71.8 ± 3.8	178.0 ± 10.8	283.8 ± 16.7	248.4 ± 17.0	9.0 ± 0.7	3.6 ± 0.1*

The results are expressed as the mean ± standard deviation. Group A: control; Group B: dioxins + DL-PCBs; Group C: curcumin intervention; ^#^*p* < 0.05 vs. group A (Q test); *p < 0.05 vs. group B (Q test).

**Figure 3:**
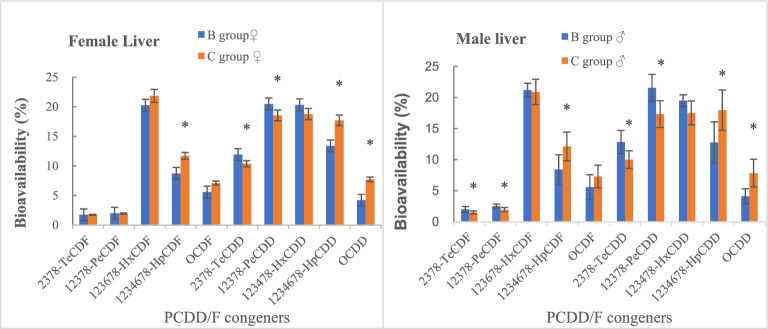
Effects of curcumin on bioavailability of PCDDF congeners (*p < 0.05) (drawn with Microsoft Excel).

There was no obvious sex difference in the bioavailability of PCDD/F congeners in the livers of rats in groups B and C (*t*-test, *p* > 0.05). As compared to group B, group C exhibited significantly decreased bioavailability of 2,3,7,8-TeCDD and 1,2,3,7,8-PeCDD in the livers of female rats, as well as 2,3,7,8-TeCDF, 1,2,3,7,8-PeCDF, 2,3,7,8-TeCDD and 1,2,3,7,8-PeCDD in the livers of male rats (*t*-test, *p* < 0.05). The bioavailability of 1,2,3,4,6,7,8-HpCDF, 1,2,3,4,6,7,8-HpCDD and OCDD was significantly increased in the livers of female and male rats (*t*-test, *p* < 0.05). However, there were no significant changes in the bioavailability of other PCDD/Fs (*t*-test, *p* > 0.05). In general, hexa-chlorinated PCDD/Fs had the highest bioavailability, while that of tetra- and penta-chlorinated PCDFs had the lowest. Besides, the administration of curcumin did not alter the bioavailability patterns of different PCDD/Fs (Fig. 3).

**Figure 4:**
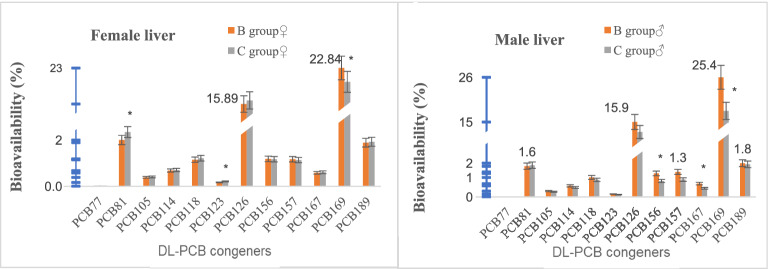
Effects of curcumin on bioavailability of DL-PCB congeners (*p < 0.05) (drawn with Microsoft Excel).

There was no obvious sex difference in the bioavailability of PCBs in the rat liver (*t*-test, *p* > 0.05). The bioavailability of PCB169 in the livers of female rats and that of PCB156, PCB167 and PCB169 in the livers of male rats was decreased significantly in group C in contrast to group B (*t*-test, *p* < 0.05). The bioavailability of PCB81 and PCB123 was significantly higher in female rats (*t*-test, *p* < 0.05). Additionally, the changes in the bioavailability of other PCBs were not significant (*t*-test, *p* > 0.05). With the exception of the high bioavailability of PCB169 and PCB126, no apparent changes in the bioavailability were found with variations in the number of substituted chlorine atoms (4–8), and the administration of curcumin did not alter the changes in bioavailability of PCBs (Fig. 4).

## Discussion

After exposure to 1 ng/mL of PCDD/Fs and 10 ng/mL of DL-PCBs, there was no significant change in the BW and liver weight of female and male rats, or in the HSI of female rats, whereas the HSI of male rats was obviously increased. After administration of curcumin, the HSI of the rats was decreased, indicating that injury to the liver may have resolved. The protective effect of curcumin has also been reported in liver injury induced by carbon tetrachloride, ischemia–reperfusion, and alcohol^10^. One study found that TeCDD toxicity varied among different species, different strains within the same species, and different sexes within the same strain^11^. Moreover, female Long–Evans rats were reportedly more sensitive to the acute lethality of TeCDD than males, while male mice and guinea pigs were more sensitive to TeCDD toxicity than females^12,13^. In the present study, the male rats were more sensitive to the hepatotoxicity of POPs with a diphenyl ring, which is inconsistent with previous reports in the literature. A possible explanation for this discrepancy is that the SD rats were used as the laboratory murine strains and exposed to a low-concentration mixture (10 types of PCDD/Fs and 12 types of PCBs) for 28 days, which is different from previous studies; thus, the sex-dependent toxic effect may have differed.

The administration of curcumin markedly decreased the bioavailability of WHO-PCDD/F-TEQ, WHO-DL-PCB-TEQ, and total TEQ in the liver of male rats, but only slightly reduced the bioavailability of WHO-PCDD/F-TEQ in the liver of female rats, indicating that curcumin can attenuate the bioavailability of DL-POPs. These results suggest that the decrease in the bioavailability of DL-POPs may be a detoxification mechanism of curcumin.

Following administration of curcumin, the bioavailability of tetra- and penta-chlorinated PCDDs (including 2,3,7,8-TeCDD and 1,2,3,7,8-PeCDD, with a TEF of 1) and tetra- and penta-chlorinated PCDFs (including 2,3,7,8-TeCDF and 1,2,3,7,8-PeCDF, with a TEF of 0.1 and 0.03, respectively) was reduced markedly in male rats, while the impact on the bioavailability of WHO-PCDD/F-TEQ based on wet weight was greater. Meanwhile, there was no significant change in the bioavailability of hexa-chlorinated PCDD/F and OCDF (8Cl). The bioavailability of hepta-chlorinated PCDD/F (TEF = 0.01) and OCDD (TEF = 0.0003) was increased, which had a small influence on the bioavailability of WHO-PCDD/F-TEQ. In addition to no obvious changes in the bioavailability of tetra- to hexa-chlorinated PCDFs, variations in bioavailability of the remaining PCDD/Fs in female rats were similar to those in male rats. The bioavailability of WHO-PCDD/F-TEQ was decreased in female and male rats, illustrating that curcumin can decrease the bioavailability of PCDD/Fs. The chemical structure (diphenyl structure) of curcumin resembles that of PCDD/Fs and may competitively repress the absorption of PCDD/Fs in the epithelial cells of the small intestine. Nevertheless, the specific mechanism of curcumin in lowering the bioavailability of PCDD/Fs remains unclear and needs to be verified. A previous study revealed that curcumin can inhibit the absorption of other chemical substances, such as cholesterol, in the intestinal tract^14^.

In female rats, curcumin only decreased the bioavailability of PCB169 (TEF = 0.03), while the bioavailability of PCB81 and PCB123 was clearly increased, and no obvious changes in the bioavailability of the other PCBs were observed. There was no obvious change in the overall bioavailability of WHO-DL-PCB-TEQ based on wet weight. Moreover, in the liver of male rats, the bioavailability of PCB156 (TEF = 0.00003), PCB167 (TEF = 0.00003), and PCB169 was significantly decreased, while that of PCB126 (TEF = 0.1) was slightly decreased, but not significantly. For other PCBs, changes in bioavailability were not apparent and the bioavailability of WHO-DL-PCB-TEQ based on wet weight was reduced, implying that there is a significant sex difference in the intervention effect of curcumin on the bioavailability of PCBs. As typical endocrine-disrupting chemicals with estrogen and antiandrogen effects^15^, PCBs can affect the function of the hypothalamus–pituitary (HP) axis and hinder the synthesis, metabolism, and action of hormones^16^. The decreased bioavailability of PCBs in males may be related to the functional change of the HP axis, which needs to be confirmed. The bioavailability of PCDD/Fs and PCBs had independently changed along with the increase in number of chlorine atoms in the molecular structure. Specifically, tetra- and penta-chlorinated PCDFs had the lowest bioavailability, while hexa-chlorinated PCDD/Fs had the highest, and that of PCBs displayed no obvious change. Nevertheless, the changes to those indicators were not altered by curcumin administration.

Distribution of PCDD/Fs in tissues is primarily dependent on the structure, chlorination level, dose range, absorption, metabolism, and transport in blood of homologs^17^. Kim et al*.* applied the quantitative structure–property relationship method to predict the physicochemical properties of PCDD homologs^18^, which revealed that as the number of substituted chlorine atoms was increased, the water solubility of PCDDs was gradually weakened, while the octanol–water partition coefficient (*K*_OW_, log*K*_OW_ is a substitute index of lipid–water partition coefficient) had gradually increased, indicating that highly chlorinated PCDDs are preferentially absorbed in the small intestine, and thus the bioavailability of these compounds is relatively high in the liver. However, the preferential absorption of more chlorine atoms is limited over a certain range because the organic pollutants with too high log*K*_OW_ and too low water solubility are not conducive to absorption by cells. Moreover, the bioavailability of OCDD and OCDF (8Cl) in the liver and kidney fat of sheep is low^19^. Budinsky et al*.* reported that the bioavailability of 2,3,7,8-TeCDF and 1,2,3,7,8-PeCDF is low, and that of 1,2,3,6,7,8-HxCDF is high in the rat liver, which is in line with the results of our study, although the bioavailability of 2,3,4,7,8-PeCDF was higher^20^. However, the reason for the difference in the bioavailability of 1,2,3,7,8-PeCDF and 2,3,4,7,8-PeCDF remains unclear, as both are pentachlorides. Kim et al*.* also demonstrated that PCDD homologs have the same number of chlorine atoms, but the substituted positions and *K*_OW_ differed^18^. It may be that furans have similar conditions. Previous studies have reported that PCDD/F levels in deer tissues were almost two-fold greater than in wild boar tissues, and the bioaccumulative coefficients (the ratio of the concentration in the muscle to the concentration in the environmental samples) in both deer and wild boar decreased with an increase in the number of chlorine atoms in PCDD/F molecules^21^. Such an accumulation mode of PCDD/F homologs differs from that in the present study, suggesting that there are differences in the absorption modes of PCDD/Fs among various species and between low-level exposure in the field and high-level exposure in the laboratory.

A previous study reported no obvious change in the bioavailability of PCBs in wild boar and deer^21^. However, Zhang et al*.* utilized leaches in rice paddy fields to investigate the bioaccumulation of organochlorine pesticides and PCBs, and found that the bioaccumulation was affected by environmental factors (temperature, soil, etc.), biological factors (species), and chemical factors^22^. In the majority of bioaccumulation models, the bioaccumulation factors of organochlorine compounds are often associated with *K*_OW_^23^. When log*K*_OW_ is increased from 6.0 to ~ 7.0, the bioaccumulative coefficient of PCBs increases, but decreases conversely as log*K*_OW_ increases. Moreover, it has been observed that PCB156 and PCB157 have the highest accumulative coefficients^22^. It can be seen that the bioavailability may vary from species to species.

There were some limitations to this study that should be addressed. First, the number of animals in each group was small, and the individual differences were large, so it was difficult to obtain significant differences. Second, the bioavailability of PCDD/Fs and PCBs was only examined in the liver, while contents in other tissues and excretion in urine and feces were not tested. Third, not all PCDD/Fs and PCBs were investigated, and thus, the effects of curcumin on the bioavailability of PCDD/Fs and PCBs could not be comprehensively assessed. The above limitations need to be improved by subsequent studies.

## Conclusion

Curcumin did not alter the bioavailability of various PCDD/Fs and DL-PCBs, but significantly reduced that of WHO-PCDD/F-TEQ based on wet weight in female and male rats. These findings illustrate that curcumin can decrease the bioavailability of PCDD/Fs, suggesting that curcumin competitively inhibits the absorption of PCDD/Fs in the epithelial cells of the small intestine due to the similar chemical structure (diphenyl structure) between curcumin and PCDD/Fs. As a result, the bioavailability of PCDD/Fs is decreased. Furthermore, curcumin reduced the bioavailability of WHO-DL-PCB-TEQ in the livers of male rats, but not female rats. Such an apparent sex difference may be correlated with functional changes to the HP axis. Curcumin can trigger evident drops in the bioavailability of PCDD/Fs and PCBs, suggesting that the decrease in the bioavailability of DL-POPs is one of the detoxification mechanisms of curcumin.

## Supplementary Information

Supplementary Tables.
