# Supplementary material for: Effects of curcumin on the bioavailability of dioxin-like pollutants in rats
Source: Sci Rep. 2021 Jun 14;11:12461. doi: 10.1038/s41598-021-92085-3 (PMC8203761; doi:10.1038/s41598-021-92085-3)
Supplement: Supplementary file 1 — Supplementary Tables. [file 41598_2021_92085_MOESM1_ESM.docx]

| ***S*-1**, **PCDD/F and DL-PCB congeners** | | |
| --- | --- | --- |
|  | Congeners | Toxic Equivalent Factor (TEF) |
| PCDD/Fs | 2,3,7,8-TeCDF | 0.1000 |
|  | 1,2,3,7,8-PeCDF | 0.0300 |
|  | 1,2,3,6,7,8-HxCDF | 0.1000 |
|  | 1,2,3,4,6,7,8-HpCDF | 0.0100 |
|  | OCDF | 0.0003 |
|  | 2,3,7,8-TeCDD | 1.0000 |
|  | 1,2,3,7,8-PeCDD | 1.0000 |
|  | 1,2,3,4,7,8-HxCDD | 0.1000 |
|  | 1,2,3,4,6,7,8-HpCDD | 0.0100 |
|  | OCDD | 0.0003 |
| DL-PCBs | PCB77 | 0.0001 |
|  | PCB81 | 0.0003 |
|  | PCB105 | 0.00003 |
|  | PCB114 | 0.00003 |
|  | PCB118 | 0.00003 |
|  | PCB123 | 0.00003 |
|  | PCB126 | 0.1 |
|  | PCB156 | 0.00003 |
|  | PCB157 | 0.00003 |
|  | PCB167 | 0.00003 |
|  | PCB169 | 0.03 |
|  | PCB189 | 0.00003 |
| TEQ _PCDD/F_ | / | 2.3506 |
| TEQ _DL-PCB_ | / | 0.13064 |
| TEQ _PCDD/F+DL-PCB_ | / | 2.48124 |

| ***S-*2*,* Daily exposure information** | | | | | | | | | | | | | | | | | | |
| --- | --- | --- | --- | --- | --- | --- | --- | --- | --- | --- | --- | --- | --- | --- | --- | --- | --- | --- |
|  | **A ♀** | | | **A♂** | | | **B♀** | | | **B♂** | | | **C♀** | | | **C♂** | | |
| **Day** | **mL*** | **PCDD/F** | **DL-PCB** | **mL*** | **PCDD/F** | **DL-PCB** | **mL#** | **PCDD/F TEQ (ng)** | **DL-PCB TEQ (ng)** | **mL#** | **PCDD/FTEQ**  **(ng)** | **DL-PCB TEQ (ng)** | **mL**  **#** | **PCDD/FTEQ**  **(ng)** | **DL-PCB TEQ**  **(ng)** | **mL**  **#** | **PCDD/F TEQ**  **(ng)** | **DL-PCB TEQ**  **(ng)** |
| 1 | 0.35 | / | / | 0.36 | / | / | 0.36 | 0.85 | 0.47 | 0.36 | 0.86 | 0.48 | 0.35 | 0.83 | 0.46 | 0.36 | 0.84 | 0.47 |
| 2 | 0.39 | / | / | 0.40 | / | / | 0.40 | 0.93 | 0.52 | 0.40 | 0.95 | 0.53 | 0.39 | 0.92 | 0.51 | 0.40 | 0.94 | 0.52 |
| 3 | 0.42 | / | / | 0.43 | / | / | 0.42 | 0.99 | 0.55 | 0.43 | 1.02 | 0.57 | 0.42 | 1.00 | 0.55 | 0.43 | 1.02 | 0.57 |
| 4 | 0.45 | / | / | 0.47 | / | / | 0.46 | 1.08 | 0.60 | 0.48 | 1.13 | 0.63 | 0.45 | 1.07 | 0.59 | 0.47 | 1.11 | 0.61 |
| 5 | 0.49 | / | / | 0.49 | / | / | 0.50 | 1.18 | 0.66 | 0.51 | 1.20 | 0.67 | 0.49 | 1.15 | 0.64 | 0.50 | 1.18 | 0.66 |
| 6 | 0.51 | / | / | 0.53 | / | / | 0.52 | 1.23 | 0.68 | 0.54 | 1.28 | 0.71 | 0.53 | 1.24 | 0.69 | 0.52 | 1.23 | 0.69 |
| 7 | 0.55 | / | / | 0.57 | / | / | 0.56 | 1.31 | 0.73 | 0.59 | 1.38 | 0.76 | 0.54 | 1.28 | 0.71 | 0.57 | 1.34 | 0.74 |
| 8 | 0.58 | / | / | 0.61 | / | / | 0.60 | 1.41 | 0.78 | 0.62 | 1.45 | 0.81 | 0.58 | 1.37 | 0.76 | 0.60 | 1.41 | 0.78 |
| 9 | 0.61 | / | / | 0.64 | / | / | 0.62 | 1.46 | 0.81 | 0.65 | 1.53 | 0.85 | 0.61 | 1.44 | 0.80 | 0.64 | 1.51 | 0.84 |
| 10 | 0.65 | / | / | 0.68 | / | / | 0.66 | 1.55 | 0.86 | 0.69 | 1.63 | 0.91 | 0.64 | 1.51 | 0.84 | 0.68 | 1.60 | 0.89 |
| 11 | 0.66 | / | / | 0.73 | / | / | 0.68 | 1.60 | 0.89 | 0.74 | 1.74 | 0.97 | 0.66 | 1.56 | 0.87 | 0.72 | 1.69 | 0.94 |
| 12 | 0.71 | / | / | 0.77 | / | / | 0.73 | 1.71 | 0.95 | 0.78 | 1.82 | 1.01 | 0.70 | 1.64 | 0.91 | 0.77 | 1.81 | 1.01 |
| 13 | 0.72 | / | / | 0.81 | / | / | 0.75 | 1.76 | 0.98 | 0.82 | 1.92 | 1.07 | 0.72 | 1.69 | 0.94 | 0.80 | 1.87 | 1.04 |
| 14 | 0.75 | / | / | 0.85 | / | / | 0.76 | 1.79 | 0.99 | 0.86 | 2.03 | 1.13 | 0.74 | 1.75 | 0.97 | 0.85 | 2.01 | 1.12 |
| 15 | 0.79 | / | / | 0.90 | / | / | 0.79 | 1.86 | 1.03 | 0.91 | 2.14 | 1.19 | 0.77 | 1.82 | 1.01 | 0.90 | 2.12 | 1.18 |
| 16 | 0.80 | / | / | 0.91 | / | / | 0.82 | 1.93 | 1.07 | 0.94 | 2.22 | 1.23 | 0.79 | 1.86 | 1.03 | 0.93 | 2.20 | 1.22 |
| 17 | 0.84 | / | / | 0.99 | / | / | 0.86 | 2.01 | 1.12 | 1.00 | 2.35 | 1.31 | 0.83 | 1.94 | 1.08 | 0.99 | 2.32 | 1.29 |
| 18 | 0.86 | / | / | 1.03 | / | / | 0.86 | 2.02 | 1.12 | 1.04 | 2.44 | 1.35 | 0.84 | 1.98 | 1.10 | 1.03 | 2.41 | 1.34 |
| 19 | 0.89 | / | / | 1.08 | / | / | 0.89 | 2.08 | 1.16 | 1.09 | 2.56 | 1.42 | 0.86 | 2.01 | 1.12 | 1.07 | 2.52 | 1.40 |
| 20 | 0.90 | / | / | 1.11 | / | / | 0.90 | 2.12 | 1.18 | 1.13 | 2.65 | 1.47 | 0.88 | 2.07 | 1.15 | 1.11 | 2.60 | 1.45 |
| 21 | 0.92 | / | / | 1.15 | / | / | 0.92 | 2.16 | 1.20 | 1.16 | 2.72 | 1.51 | 0.88 | 2.07 | 1.15 | 1.15 | 2.70 | 1.50 |
| 22 | 0.93 | / | / | 1.18 | / | / | 0.93 | 2.19 | 1.22 | 1.20 | 2.83 | 1.57 | 0.91 | 2.14 | 1.19 | 1.19 | 2.80 | 1.56 |
| 23 | 0.96 | / | / | 1.19 | / | / | 0.95 | 2.23 | 1.24 | 1.23 | 2.88 | 1.60 | 0.90 | 2.11 | 1.18 | 1.22 | 2.87 | 1.60 |
| 24 | 0.96 | / | / | 1.27 | / | / | 0.97 | 2.28 | 1.27 | 1.26 | 2.97 | 1.65 | 0.94 | 2.20 | 1.22 | 1.27 | 2.98 | 1.65 |
| 25 | 0.99 | / | / | 1.31 | / | / | 0.99 | 2.32 | 1.29 | 1.32 | 3.11 | 1.73 | 0.95 | 2.23 | 1.24 | 1.30 | 3.06 | 1.70 |
| 26 | 0.99 | / | / | 1.36 | / | / | 1.01 | 2.37 | 1.32 | 1.35 | 3.18 | 1.77 | 0.97 | 2.27 | 1.26 | 1.35 | 3.17 | 1.76 |
| 27 | 1.02 | / | / | 1.39 | / | / | 1.01 | 2.38 | 1.32 | 1.40 | 3.30 | 1.83 | 0.96 | 2.27 | 1.26 | 1.38 | 3.24 | 1.80 |
| 28 | 1.03 | / | / | 1.44 | / | / | 1.03 | 2.42 | 1.35 | 1.44 | 3.39 | 1.88 | 0.98 | 2.31 | 1.29 | 1.42 | 3.34 | 1.85 |
| **Total** | **20.73** | **/** | **/** | **24.66** | **/** | **/** | **20.94** | **49.23** | **27.36** | **24.96** | **58.66** | **32.60** | **20.30** | **47.72** | **26.52** | **24.62** | **57.88** | **32.17** |

*：The volume of soybean oil gavaged (mL）

#：The volume of polluted soybean oil gavaged (mL）

| ***S*-3, Summary of DL-POPs exposure dose** | | | | | | |
| --- | --- | --- | --- | --- | --- | --- |
| **(**$\bar{\boldsymbol{x}}$**± s)** | **A ♀** | **A♂** | **B♀** | **B♂** | **C♀** | **C♂** |
| PCDD/F TEQ (ng) | / | / | 49.2±1.9 | 58.6±1.8 | 47.7±1.3 | 57.9±3.4 |
| DL-PCB TEQ (ng) | / | / | 27.4±1.0 | 32.6±1.0 | 26.5±0.7 | 32.2±1.9 |
